# Neurology and physician-assisted suicide: position of the Italian society of neurology

**DOI:** 10.1007/s10072-025-08038-5

**Published:** 2025-02-27

**Authors:** Eugenio Pucci, Nicola Ticozzi, Giancarlo Comi, Gianluigi Mancardi, Leandro Provinciali, Alessandro Padovani, Alessandra Solari

**Affiliations:** 1 Azienda Sanitaria Territoriale Fermo, UOC Neurologia, Fermo, Italy; 2 Department of Neuroscience, IRCCS Istituto Auxologico Italiano, Milan, 20149 Italy; 3 Department of Pathophysiology and Transplantation, Università degli Studi di Milano, Milan, 20122 Italy; 4 Dipartimento di Scienze Neuroriabilitative, Casa di Cura Igea, Milan, Italy; 5 Department of Neuroscience, Rehabilitation, Ophthalmology, Genetics, Maternal and Child Health, University of Genoa, Genoa, Italy; 6 Professore Emerito di Neurologia, Università Politecnica delle Marche, Ancona, Italy; 7 Neurology Unit, Department of Clinical and Experimental Sciences, University of Brescia, Brescia, Italy; 8 Department of Continuity of Care and Frailty, Neurology Unit, ASST Spedali Civili Hospital, Brescia, Italy; 9 Neurobiorepository and Laboratory of Advanced Biological Markers, University of Brescia and ASST Spedali Civili Hospital, Brescia, Italy; 10 Brain Health Center, University of Brescia, Brescia, Italy; 11 Neuroepidemiology Unit, Fondazione IRCCS Istituto Neurologico Carlo Besta, Milan, Italy

**Keywords:** Physician-assisted suicide, Chronic neurological illness, Neuropalliative care, Quality of life, Neurology/Ethics, Euthanasia

## Abstract

This position paper explores the complex issue of Physician-Assisted Suicide (PAS) in the context of Neurology. It discusses the legal, ethical, and clinical challenges surrounding PAS, including the role of neurologists in assessing eligibility based on prognosis, decision-making capacity, and functional status. The paper outlines Italy’s legal framework regarding PAS, following a landmark 2019 Constitutional Court ruling, which permits PAS under strict conditions for patients suffering from incurable illnesses. It also compares PAS regulations in other European and non-European countries. The Italian Society of Neurology (SIN) emphasizes the importance of respecting patient autonomy while advocating for comprehensive palliative care (PC). The SIN’s position is that PAS should be legalized under specific circumstances but insists on ensuring equitable access to PC services before considering PAS. Additionally, the SIN supports the training of neurologists in palliative and end-of-life care, underlining the need for careful monitoring and regulation of PAS practices to prevent potential ethical and legal abuses.

**Supplementary Information:**

The online version contains supplementary material available at 10.1007/s10072-025-08038-5.

## Introduction

The progress of biomedical technologies has expanded the boundaries of care, offering treatment options to patients who until a few years ago were considered incurable and prolonging survival even in conditions of great suffering and dependence. *Physician-Assisted Suicide* (*PAS*) is a complex and sensitive topic, often divisive depending on where one stands among the different ethical positions. This document aims to elicit a reflection on the theme of *PAS* in the context of Neurology. Such a reflection requires precise terminology to ensure clear and productive discourse; a glossary of definitions has been produced on this regard, which is aligned with most recent international consensus documents and with the Italian regulatory frameworks [1]. These definitions are identified in italics in this manuscript. For *PAS*, we adopted the definition of the European Association for Palliative Care (EAPC): ”a physician intentionally helping a patient to terminate life by providing drugs for self-administration, at that person’s voluntary and competent request” [2].

The regulatory context related to *PAS* in Italy will be considered and compared to other European and non-European countries. Some aspects that make *PAS* relevant in many neurological conditions will be addressed, in particular disease prognosis, *decision-making capacity* and functional capacity. Finally, the position of the Italian Society of Neurology (SIN) concerning *PAS* will be illustrated. This position paper was developed as outlined in the accompanying glossary [1].

An Italian version is available as a Supplementary File.

## The Italian context

In Italy, assisted suicide is a crime punishable by up to 12 years’ imprisonment, according to article 580 (instigation or assistance to suicide) of the Codice Penale (Penal Code) [3]. Judgment 242/2019 of the Italian Constitutional Court (ICC) has placed an exception to punishability in so far as the Penal Code “does not exclude the punishment of those who […] facilitates the fulfilment of the autonomously and freely formed intent to commit suicide of a person fully capable of making free and informed decisions kept alive by *life-support treatments* (*LSTs*) and suffering from an incurable illness which is a source of physical or psychological suffering that he or she considers intolerable, [Authors’ note: *total pain*] provided that these conditions and the method of implementation have been verified by a public national health system facility after consulting the territorially competent ethics committee.” [4]. It is required that the patient’s will has been clearly and unequivocally expressed, compatibly with what is permitted by their conditions, and that the patient has been adequately informed about *PAS* and all the other treatment options, specifically *palliative care* (*PC*) and, where appropriate, *palliative sedation*.

The verification of the four *PAS* requirements (patient’s preserved *decision-making capacity*, intolerable physical or psychological suffering, *unfavourable prognosis*, and presence of *LST*) is certified by an interdisciplinary commission of employees of a public national health system facility. The resulting document is submitted to the territorially competent ethics committee for non-binding opinion.

Although the ICC, in Judgment no. 135/2024, deemed it reasonable and consistent with the Italian Constitution to restrict the non-punishability of assisting suicide to cases where the patient is dependent on *LSTs*, it clarified the requirement of “dependence on *LSTs*” placing it within a broader context, regardless of its technical complexity, invasiveness, or the direct involvement of health professionals [1].

In the absence of a specific legislation, the ICC judgments lay the foundation for the current access to *PAS*, which is far from being clarified, with procedures still inconsistent across the national territory.

## The role of the neurologist

The role of the physician (the neurologist as far as neurological disease is concerned) in cases of *PAS* is multifaceted, involving clinical, ethical, and legal issues.

Regarding the clinical role, the physician (working within a public national health system facility) may be involved in assessing the patient’s condition and eligibility according to Judgment 242/2019 of the ICC [4]. This includes confirming the diagnosis of “an incurable illness which is a source of physical or psychological suffering” that the patient “considers intolerable”. The physician must also understand the patient’s prognosis and verify that their suffering cannot be alleviated by other means. Additionally, it is essential to assess whether the patient “is fully capable of making free and informed decisions”, and is ”kept alive by *LSTs*”. Finally, it is the physician who can assess whether the patient would be able to self-administer the lethal drug, which eventually requires the maintenance of motor and swallowing capacity. Given that mental disturbances and/or psychological distress and/or cognitive deficit can challenge the decision-making process, it is advisable for the neurologist to collaborate with other specialists, such as psychiatrists, psychologists, or neuropsychologists, for a more comprehensive assessment.

It should be ensured that the patient fully understands the implications of their decision. This requires a thorough discussion of the patient’s condition, prognosis, and alternative options to ensure that their intent to end their own life is “autonomously and freely formed”.

The physician is not required to prescribe lethal medication unless directly involved in the *PAS* procedure on patient’s request. In fact, this responsibility falls to the patient’s designated physician, who has been entrusted with this role. The physician who assists in *PAS* implementation can be supported by other professionals for preparation of the appropriate setting, the lethal drug, and the delivery device. If consented to by the patient, the physician should strive to collaborate closely with specialist *PC* professionals, while also considering management of the bereavement phase.

The ethical role of the physician in *PAS* is complex, involving a delicate balance between respecting patient self-determination and managing professional responsibilities and personal moral beliefs. They must navigate this balance while adhering to the clinical ethical principles of autonomy, beneficence, and non-maleficence [5]. Preserving patient confidentiality is crucial. However, when legal or ethical obligations conflict, physicians should seek guidance from legal and bioethical experts. The Local ethics committees (in Italian: comitati etici locali per la pratica clinica), which are available in some Italian regions, can play a significant role in addressing these as well as other issues and conflicts [6, 7].

Engaging in *PAS* can pose professional challenges, including potential conflicts with personal moral beliefs or professional ethical convictions. Some physicians may experience moral distress or face criticism from colleagues or the public.

Physicians must comply with specific legal requirements, such as documenting the patient’s request, ensuring that all the eligibility criteria are met, and reporting the case to appropriate authorities. This process must be meticulously documented to avoid legal challenges.

The practice of *PAS* affects the broader medical community’s perception of the role of physicians.

Currently, Italian physicians are guided by the ethical code established by the National Federation of Orders of Surgeons and Dentists (FNOMCEO), which states that “the physician, even at the patient’s request, must neither perform nor facilitate acts aimed at causing the patient’s death” (Article 17 of the Code of Medical Ethics) [8]. However, following the ICC Judgment 242/2019 [4], there is an ongoing debate about the implications for Article 17 of the Code of Medical Ethics. To reconcile the Code with new legal realities, an amendment has been introduced, stating that “the physician’s free choice to assist, based on the principle of individual self-determination, in the intent to commit suicide […] must always be evaluated on a case-by-case basis and entails […] non-punishability from a disciplinary perspective” [8].

The disciplinary councils of the medical associations will be called upon to evaluate each case specifically, to ensure that all the conditions provided for by the ICC’s ruling are met.

The FNOMCEO has therefore decided to leave colleagues free to act according to the law and their own conscience. Even if the principles of Article 17 remain unchanged, the amendment indicates a shift in the absolute prohibition of acts causing death at the patient’s request. This is in line with the ICC provisions which, outside the delimited area, reiterated that “criminalising assistance to suicide is not per se unconstitutional […] rather, such criminalisation is justified by the need to protect the right to life, especially of the weakest and most vulnerable persons [ensuring that] the legal system seeks to protect these persons by preventing external parties from interfering in a decision as extreme and irreparable as suicide” [9].

The adaptation of this ethical framework to ongoing legal changes is a fundamental area of discussion within the Italian medical community. In the current situation, the ICC Judgement n. 242/2019 [4] states that there is no “obligation for physicians” to assist in *PAS*, leaving it as a matter of individual conscience for physicians to decide whether to comply with a patient’s request. Since *PAS* does not impose any obligations on healthcare professionals, it should not be necessary to establish conscientious objection. However, the ICC recognized that public healthcare institutions are obligated to respond to *PAS* requests. There is no issue when a neurologist voluntarily agrees to participate in *PAS* within the patient-physician relationship. The challenge arises when a neurologist is required to participate in *PAS* procedures mandated by their public institution, such as serving on a committee to determine eligibility for *PAS*. In such cases, the neurologist may invoke the “conscience clause,” which allows them to object based on constitutional principles and ethical codes in situations not regulated by the law. However, if no public healthcare physician is willing to participate, an unsustainable situation would arise, hindering the patient’s right to *PAS.* The Italian National Bioethics Committee (CNB) [10] has emphasized that conscientious objection must be exercised in a sustainable way, ensuring that services are available to uphold patients’ rights despite objections. This principle could be similarly applied to the “conscience clause.”

## The international context

As from a recently published review [11], in Switzerland, the Netherlands and some US states, a process of care that leads to death occurs in neurological patients at a frequency lower only than cancer patients. In the studies examined, the most frequent neurological condition was dementia, and *euthanasia* was 10 times more frequent than *PAS*.

The Table illustrates the regulation of *PAS* in the main European and non-European countries. The main eligibility criteria are considered, including those provided for by the current regulation of *PAS* in Italy.

**Table 1:** The regulation of physician-assisted suicide (PAS) in the main European and non-European countries. The main policy criteria are considered, including those provided for by the current Italian regulation.

Country	Law	Year	Policy criteria							
			Age (years)	Willingness	Decision-making capacity	Condition	Unfavorable prognosis	LSTs	Mandatory reflexion period	Second evaluation/ Commission
***EUROPE***										
Switzerland^a,b^	No	1942	≥ 18	Yes	Yes	NS, including psychiatric disorder	NS	NS	NS	NS/NS
Netherlands^c^	*Termination of Life Upon Request Act*	2002	≥ 12 (parental consent 12–16)	Yes	Yes, at the time of request, including ACP	NS, including psychiatric disorder	NS	NS	NS	Yes (psychiatric disorder)/Yes
Belgium^a,c,d,^	No	2002	Any	Yes	Yes, at the time of request, including ACP	NS, including psychiatric disorder	NS	NS	NS	Yes (psychiatric disorder)/Yes
Luxembourg^c^	*Right to Die with Dignity*	2009	≥ 16 (Parental consent 16–18)	Yes	Yes, at the time of request, including ACP	NS, including psychiatric disorder	NS	NS	NS	Yes (psychiatric disorder)/Yes
Italy^a^	No	2019 CC	≥ 18	Yes	Yes	Irreversible pathology and source of intolerable physical or psychological suffering	NS	Yes	NS	Yes/Yes
Germany^a^	No	2020 CC	≥ 18	Yes	Yes	NS, excluding acute psychiatric disorder	NS	NS	NS	NS
Spain^c^	Yes	2021	≥ 18	Yes	Yes	Serious or incurable illness or a chronic or incapacitating condition that causes intolerable suffering	NS	NS	15 days written	Yes/Yes
***AMERICAS***										
Oregon Washington D.C. Maine	*Death with Dignity Act*	1994 2009 2016 2019	≥ 18	Yes	Yes	Terminal	< 6 months	NS	2 days written 15 days oral	Yes/NS
Montana^a^	No	2009	NS	Yes	Yes	NS	NS	NS	NS	NS/NS
Vermont	*Patient Choice and Control at the EOL Act*	2013	≥ 18	Yes	Yes	Terminal	< 6 months	NS	2 days written 15 days oral	Yes/NS
California Colorado New Mexico	*End of Life Option Act*	2015 2016 2021	≥ 18	Yes	Yes	Terminal	< 6 months	NS	15 days oral	Yes/NS
Hawaii	*Our Care*,* Our Choice Act*	2019	≥ 18	Yes	Yes	Terminal	< 6 months	NS	6 days oral and written	Yes/NS
New Jersey	*Aid in Dying for the Terminally Ill Act*	2019	≥ 18	Yes	Yes	Terninal	< 6 months	NS	15 days oral and written	Yes/NS
Canada^c^	*Medical Assistance in Dying*	2016 (revised 2021)	≥ 18	Yes	Yes at the time of request, including ACP	Severe, with poor prognosis (psychiatric disorder under consideration)	Reasonably foreseeable	NS	10 days written 90 days if ‘reasonably foreseeableddeath’ absent (e.g., psychiatric disorder)	Yes/NS
Colombia^a,c^	No	1997^2^ 2022 Supreme Court	≥ 7 (parental consent 7–12)	Yes	Yes	Terminal or severely dependent	NS	NS	NS	NS/Yes
***OCEANIA***										
Victoria Western Au Tasmania^c^	*Voluntary Assisted Dying Act*	2017 2019 2021	≥ 18	Yes	Yes	Terminal	< 6 months (< 12 months neurodeg. disease)	NS	9 days written	Yes/Yes
New Zealand^c^	*End of Life Choice Act*	2020	≥ 18	Yes	Yes	Terminal	< 6 months	NS	No	Yes/Yes

ACP, advance care planning; CC, Constitutional Court; EOL, end of life; LST, life-sustaining treatment; NS, not specified

^a^ In these countries there is no law for PAS, but it is decriminalised

^b^ The only country where PAS is also allowed for foreign citizens

^c^ In these countries euthanasia is legal

^d^ Since 2015 extended to minors; parental consent and psychological assessment for ‘non-emancipated’ minors

As an example of the continuous evolution of the regulatory framework, reflecting changes in ethical sensitivity regarding *end-of-life* (*EoL*) issues across different countries, it is worth mentioning the developments in France and the United Kingdom (UK), where legislative processes on *EoL* issues are ongoing. In France, in April 2023, the majority position of a citizens’ assembly (“Convention Citoyenne sur la fin de vie”) supported the need to implement both *PAS* and euthanasia, arguing that neither *PAS* alone nor euthanasia alone addresses all the situations encountered [12].

In November 2024, the UK House of Commons voted to support the “Terminally Ill (End of Life) Bill”, which aims to legalize assisted dying. However, the bill still needs to pass through the House of Lords, and there are several parliamentary hurdles it must clear before it can become law [13].

## Physician-assisted suicide and neurological diseases

Several special issues arise for patients who request *PAS* because of a neurologic condition with an *unfavourable prognosis* as well as for persons for whom a non-neurologic terminal illness (e.g., cancer) forms the basis of their request but who are simultaneously living with a serious neurologic condition. Individuals diagnosed with neurodegenerative diseases may be interested in and may sometimes request *PAS* but they face difficulty with respect to timing. In fact, the average life expectancy after symptom onset may be as long as 15 to 30 years, and by the time condition fulfils criteria for request for *PAS*, severe psychiatric and cognitive symptoms often develop which can undermine the individual’s *decision-making capacity*.

In conditions that compromise *decision-making capacity* and, consequently, the possibility of providing consent, there is no possibility of requesting *PAS* (pursuant to ICC Judgement 242/19) and other *EOL* decisions (*non-treatment decisions*; *deep continuous palliative sedation*) pursuant to Italian Law 219/2017 [1].

Provision of *PAS* within the framework of *advance care planning (ACP)* is not allowed under the current Italian legislation. However, it may be possible by referring to health care facilities in foreign countries. For this reason, *ACP* should be particularly recommended at a relatively early stage in patients with diseases characterized by progressive cognitive decline. In this case, it is possible to subsequently resort to *EOL* decisions at a stage in which current consent cannot be expressed. This future *EOL* decision might include *PAS*, in agreement with EAN consensus documents and guidelines on *ACP* which suggests neurologists encouraging patients to discuss their wishes regarding *voluntary assisted death* (*VAD*) [14–16].

The American Academy of Neurology (AAN) [17] after a two-year discussion, has retracted its 1998 position, which included the “vigorous opposition of its members to participation in any form of assisted suicide”. In addition, the AAN identified the possibility of positions for or against *PAS* practice, at the discretion of the neurologist. The AAN [18] emphasized the importance of encouraging the neurologist to provide *advance directives*.

*PAS* requires self-administration, which can be problematic in many neurological diseases due to motor and swallowing impairment, producing a tension with disability laws that mandate assistance and equal access to health care and self-determination [19].

## The position of the SIN

The position of the SIN agrees with the Mental Health Action Plan 2013–2020 adopted by the World Health Organization [20]. This action plan outlines suicide prevention as a priority, with the global target of reducing the rate of suicide in countries by 10% by 2020. In the Sustainable Development Goals for 2030, suicide is a proposed indicator for health target which is to reduce premature mortality from noncommunicable diseases through prevention and treatment and promote mental health and well-being [21]. The SIN emphasizes the need for improving the care of patients suffering from life-threatening illnesses across different stages of disease by ensuring neurologists master the interdisciplinary/interprofessional aspects of palliative medicine [Box]. Patients with severe pain and/or other significant symptoms benefit from *PC* [14]. Further, it is also relevant that legalization of *PAS* should occur only after ensuring universal access to *PC* services and appropriate medications, including opioids for pain and dyspnoea [22]. When *PC* is offered with adequate treatment the desire for death may wane [10]. Optimizing *PC* will of course not eliminate all requests for *PAS* but might identify and target what can be modified, including symptoms, place of care, place of death, maximizing patients’ autonomy and *quality of life* whatever their functioning level [17].

The SIN has not expressed a specific indication regarding *PAS* in the published Code of Professional Conduct [23]. However, it is stated that the patient’s wishes must be respected regarding approaches aimed at prolonging survival and possibly ensuring a “comfortable and dignified death”.

In fact, SIN has approved its own Code of Ethics [24], which also identifies relations with patient associations and families, which are respected by a supervisory body, and refers to the aforementioned Code of Professional Conduct for Neurologists.


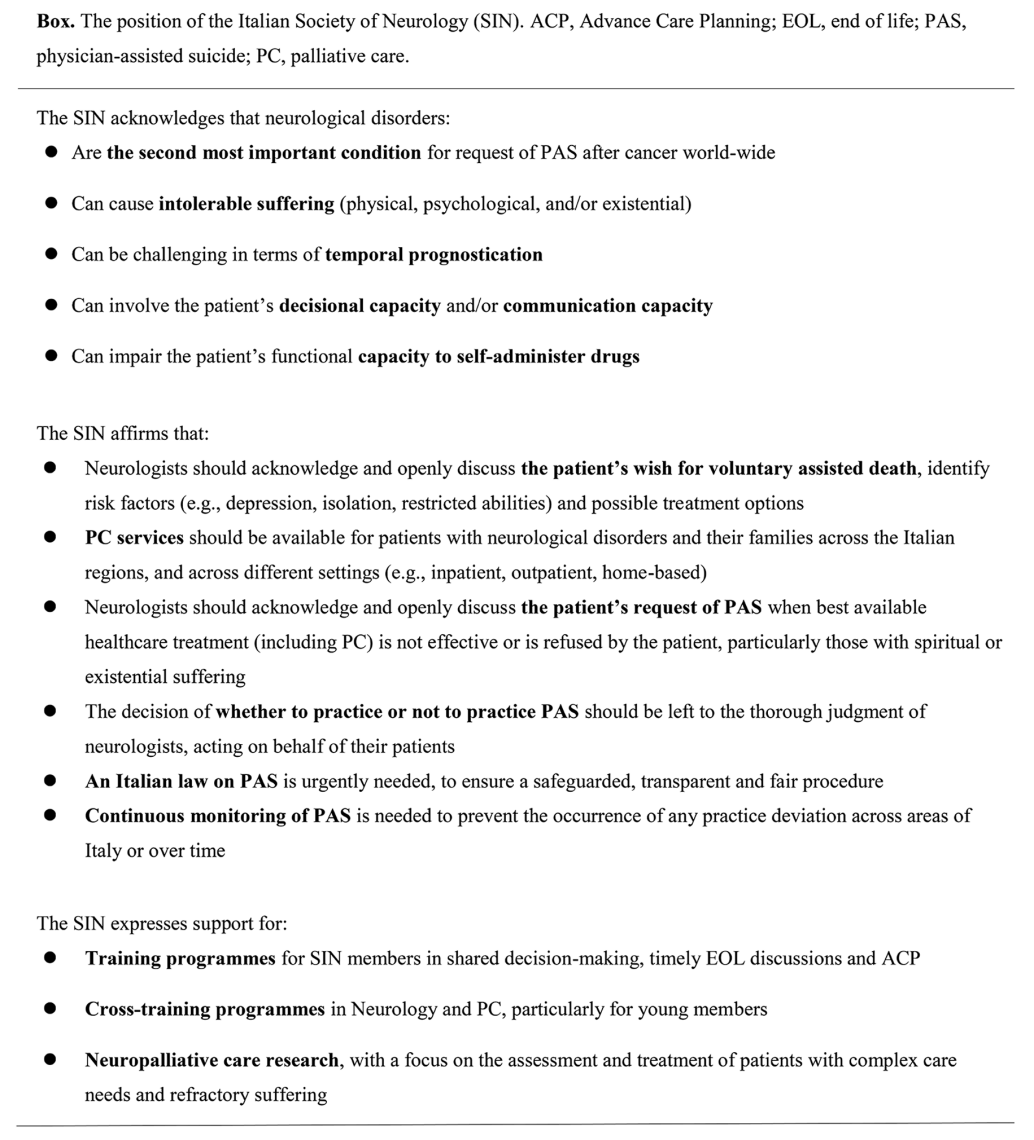


As part of the objectives to be implemented in the conduct of care, we recommend that neurologists propose *ACP* to their decision-making capable patient, able to express their wishes. Given the increasing number of neurological patients who have requested *PAS*, the SIN has planned to address the issue to increase the awareness within the neurological community and to harmonize technical and operational procedures.

In the presence of refractory symptoms, the neurologist can contribute to (but also carry out autonomously) *deep continuous palliative sedation*, with the patient’s consent. Administering *palliative sedation* can be the responsibility of neurologists if they consider themselves competent to do so, provided they receive any necessary training, have an ad hoc setting, and have the possibility to seek assistance from other specialists, primarily *PC* physicians.

Some progressive neurological diseases with *unfavorable prognosis*, as well as conditions of severe and unamendable damage to the nervous system that require continuous assistance to ensure vital functions, represent situations in which patients can consciously ask for *PAS* due to intolerable and persistent physical and/or psychological suffering (*total pain*).

In cases of *PAS* requested by neurological patients, a standardised neurological evaluation is fundamental to define conditions of irreversible damage to the nervous system (“incurable illness” in the ICC Judgement 242/19) [4]. However, the neurologist’s assessment should be carried out within an interdisciplinary and interprofessional team, comprising experts in *PC*, psychology, neuropsychology, psychiatry, anaesthesiology-resuscitation, neurorehabilitation, and the patient’s General Practitioner or equivalent.

In most progressive and irreversible neurological disease candidate for *PAS*, the identification of *LST* appears a very complex process [1]. The neurologist should be called upon to give their judgment on the presence and significance of means that may be interpreted as *LSTs*. In the face of severe or extremely severe neurological disabilities, neurologists should assess whether the assistance provided by the caregiver for the fulfilment of the patient’s vital needs should be considered *LST*.

Considering the different conditions of neurological damage, impairment of cognitive functions can also occur, such as to apparently compromise the patient’s *decision-making capacity*. In the latter conditions, the reference to *ADs* can offer the physician the awareness of a sufficient decisional capacity of the patient at the time of the expression of their wishes.

The search for reference standards for evaluating capacity in the context of *PAS* should be a goal to be pursued within the neurological scientific community.

The use of *ADs* in the context of *PAS*, far from being feasible in the current scenario, should be a point of clinical ethics debate within the SIN for the future evolution of Italian deontology and law considering what has already happened in other countries. Similarly, the debate on *euthanasia* should be a matter of discussion, particularly for the specific conditions in which severe neurological conditions of the patient make the practical implementation of suicidal conduct impossible or dangerous [19].

Training for neurologists on *PC* and *EOL* discussions should be a priority [Box], considering the current need in the graduate and postgraduate curriculum [25].

In Italy, similarly to what has been done by other scientific communities, pending the issuance of a specific legislative measure requested by the ICC, neurologists should make decisions on *PAS* related to the individual sensitivity of each person and to the provisions issued by the competent bodies activated in each individual case. In accordance with the opinion expressed by the CNB [10] and other international positions, it is believed that such legislative intervention should include a provision for conscientious objection, allowing neurologists to be exempt from carrying out procedures and activities related to *PAS*, but not from providing care prior to it. Public or affiliated healthcare institutions should be required to ensure that the procedures outlined in such a hypothetical law are carried out. To this end, it could be hope that these procedures will be included in the Italian Essential Levels of Care (Livelli Essenziali di Assistenza - LEA) [26] and exclusively held by the public healthcare system. This would ensure non-discrimination in access to *PAS* and equality across the entire national territory.

Regarding the setting in which the final act of *PAS* should take place, the proposal is primarily for the patient’s home, or alternatively, in designated settings within public or affiliated facilities, distinct from those where other diagnostic and treatment activities occur. These options should be discussed with the patient. The medications, as well as the devices necessary for self-administration (which must be adapted to the ability of each patient), should be fully covered by the national health system.

Given our position on the need for legislation, we would like to finally focus on the “slippery slope argument”, cited among other sources by the CNB [10], which we perceive as a possible paradoxical obstacle to the very definition of a law on *VAD*. The “slippery slope” metaphor highlights the risk that legislation permitting *PAS* in specific, well-defined cases might expand beyond its original intent. While acknowledging these concerns, we also worry that this argument might be a specious obstacle to patient self-determination. We strongly agree that it can be valuable if it promotes caution in establishing strict conditions for *PAS* and in introducing methods that can ensure the correctness of any *ADs* [Box]. To this, it would be advisable to establish specific committees to conduct regular reviews, monitor practices to ensure compliance with criteria for *PAS*, report any problems or abuses, and discuss potential future modifications in light of new situations.

## Concluding remarks

While the social and political debate around *VAD* remains harsh and unremitting, legislation for *VAD* is still missing in Italy, at variance with its expansion in Europe, America and Australia. Many communities are currently grappling with issues related to *EOL* care and new *VAD* legislation has been proposed by different political parties and associations. With demographic, cultural and societal trends leading to increased awareness about autonomy and self-determination at the *EOL*, *VAD* will continue to grow as a critical public health issue. On this regard, the need for research into the impact on patients, physicians, health care systems and communities is becoming ever more relevant and pressing, as is the careful monitoring of adherence to substantive and procedural safeguards. Data about the practices of *VAD* are limited. Therefore, collecting reliable data to evaluate the standard of procedures and diagnostic work-up about *PAS* is mandatory. The SIN, taking into consideration the increasing number of individuals suffering from neurological conditions asking for *VAD* and the need of a shared position, has promoted an in-depth reflection within the community of its members. The SIN acknowledges that the issue of *PAS* is extremely controversial with strong opinions on both sides of the debate and argues for the need of respecting individual autonomy and compassion toward those experiencing suffering. Concurrently, SIN claims for human life and the value of forging humane communities that dignify and protect vulnerable people. Different cultures and religious values must also be considered. For the reasons articulated, we propose that *PAS* should be legalized in selected cases while investing in education, and careful monitoring and updating of the regulations could prevent any inappropriate expansion of *VAD* practices that Italian citizens might consider immoral and incompatible with the law [Box]. SIN recognizes that moral foundations may evolve in a pluralistic society and a constitutional state, where it is not personal freedoms but their limitations that must be justified, protecting both the community’s and the individual’s interests, especially for those in vulnerable conditions that hinder the formation of a conscious and authentic will. Any future expansion of access to *VAD* should be seen as a reasonable response to new clinical, social, and cultural needs, rather than an undue concession to the “slippery slope argument”.

## Electronic supplementary material

Below is the link to the electronic supplementary material.

Supplementary Material 1
