# Correction to: Neurology and physician-assisted suicide: position of the Italian society of neurology

**DOI:** 10.1007/s10072-025-08122-w

**Published:** 2025-03-24

**Authors:** Eugenio Pucci, Nicola Ticozzi, Giancarlo Comi, Gianluigi Mancardi, Leandro Provinciali, Alessandro Padovani, Alessandra Solari

**Affiliations:** 1 Azienda Sanitaria Territoriale Fermo, UOC Neurologia, Fermo, Italy; 2 Department of Neuroscience, IRCCS Istituto Auxologico Italiano, 20149 Milan, Italy; 3 Department of Pathophysiology and Transplantation, Università degli Studi di Milano, 20122 Milan, Italy; 4 Dipartimento di Scienze Neuroriabilitative, Casa di Cura Igea, Milan, Italy; 5 Department of Neuroscience, Rehabilitation, Ophthalmology, Genetics, Maternal and Child Health, University of Genoa, Genoa, Italy; 6 Professore Emerito di Neurologia, Università Politecnica delle Marche, Ancona, Italy; 7 Department of Clinical and Experimental Sciences, Neurology Unit, University of Brescia, Brescia, Italy; 8 Department of Continuity of Care and Frailty, Neurology Unit, ASST Spedali Civili Hospital, Brescia, Italy; 9 Neurobiorepository and Laboratory of Advanced Biological Markers, University of Brescia and ASST Spedali Civili Hospital, Brescia, Italy; 10 Brain Health Center, University of Brescia, Brescia, Italy; 11 Neuroepidemiology Unit, Fondazione IRCCS Istituto Neurologico Carlo Besta, Milan, Italy

**Correction to: Neurological Sciences**

**https://doi.org/10.1007/s10072-025–08038-5**

In the original version of this manuscript, there are 3 series of errors that were spotted.

1. Regarding Table 1, the “and 'tired of living'” under the “Condition” column for Switzerland, the Netherlands, Belgium, and Luxembourg should be removed.

2. The supplementary material file is not updated and should be replaced.

3. As a distinctive graphical element, the Box should be clearly separated from the text and appropriately framed.

The corrected version should be as follows.

1. The word “and 'tired of living'” has been deleted and updated.

2. The supplementary material file has been replaced and updated.

3. Box will be captured as a figure.

The original article has been corrected.

