# Supplementary material for: Neurology and physician-assisted suicide: position of the Italian society of neurology
Source: Neurol Sci. 2025 Feb 27;46(6):2371–9. doi: 10.1007/s10072-025-08038-5 (PMC12084242; doi:10.1007/s10072-025-08038-5)
Supplement: Supplementary file 1 — Supplementary Material 1 [file 10072_2025_8038_MOESM1_ESM.pdf]

SUPPLEMENTARY INFORMATION

Translation into Italian of the article 'NEUROLOGY AND PHYSICIAN-ASSISTED SUICIDE: POSITION OF THE ITALIAN SOCIETY OF NEUROLOGY'.

NEUROLOGIA E SUICIDIO MEDICALMENTE ASSISTITO: POSIZIONE DELLA SOCIETA' ITALIANA DI NEUROLOGIA

Introduzione

Il progresso delle tecnologie biomediche ha ampliato i confini dell'assistenza, offrendo opzioni di trattamento a pazienti che fino a pochi anni fa erano considerati incurabili e prolungando la sopravvivenza anche in condizioni di grande sofferenza e dipendenza. Il *suicidio medicalmente assistito* (*SMA*) è un tema complesso e delicato, spesso divisivo a seconda delle diverse posizioni etiche. Questo documento mira a suscitare una riflessione sul tema del SMA nel contesto della neurologia. Tale riflessione richiede una terminologia precisa per garantire un discorso chiaro e produttivo; a tal proposito è stato prodotto un glossario con definizioni allineate con i più recenti documenti di consenso internazionale e con i quadri normativi italiani [1]. Queste definizioni sono indicate in corsivo nel presente manoscritto.

Per il *SMA*, abbiamo adottato la definizione dell'European Association for Palliative Care (EACP): "un medico che aiuta intenzionalmente un paziente a terminare la propria vita fornendo farmaci per l'auto-somministrazione, su richiesta volontaria di una persona mentalmente capace" [2].

Il contesto normativo relativo al *SMA* in Italia verrà descritto e confrontato con quello di altri Paesi europei e non europei. Verranno affrontati alcuni aspetti che caratterizzano il *SMA* in molte condizioni neurologiche, in particolare la prognosi della malattia, la *capacità decisionale* e la capacità funzionale. Infine, verrà illustrata la posizione della Società Italiana di Neurologia (SIN) in merito al *SMA*. Il presente position paper è stato sviluppato come indicato nel glossario che lo accompagna [1].

Il contesto italiano

In Italia, il suicidio assistito è un reato punibile fino a 12 anni di reclusione, ai sensi dell'articolo 580 (istigazione o aiuto al suicidio) del Codice Penale [3]. La Sentenza 242/2019 della Corte Costituzionale (CC) ha posto un'eccezione alla punibilità nella misura in cui il Codice Penale "non esclude la punibilità di chi [...] agevola l'esecuzione del proposito di suicidio, autonomamente e liberamente formatosi, di una persona tenuta in vita da *trattamenti di sostegno vitale* [*TSV*] e affetta da una patologia irreversibile, fonte di sofferenze fisiche o psicologiche che ella reputa intollerabili [NdA: *dolore totale*], ma pienamente capace di prendere decisioni libere e consapevoli, sempre che tali condizioni e le modalità di esecuzione siano state verificate da una struttura pubblica del servizio sanitario nazionale [SSN], previo parere del comitato etico territorialmente competente" [4]. È richiesto che la volontà della persona malata sia stata chiaramente ed inequivocabilmente espressa, compatibilmente con ciò che è permesso dalle sue condizioni, e che ella sia stata adeguatamente informata sul *SMA* e su tutte le altre opzioni di trattamento, specificamente le *cure palliative* (*CP*), e dove opportuno, la *sedazione palliativa*.

La verifica dei quattro requisiti per il *SMA* (*capacità decisionale* preservata del paziente, sofferenza fisica o psicologica intollerabile, patologia irreversibile e presenza di *TSV*) è certificata da una commissione interdisciplinare

di dipendenti di una struttura pubblica del SSN. Il documento risultante è sottoposto al comitato etico territorialmente competente per un parere non vincolante.
Sebbene la Corte Costituzionale Italiana, nella Sentenza n. 135/2024, abbia ritenuto ragionevole e coerente con la Costituzione Italiana limitare la non punibilità dell'assistenza al suicidio ai casi in cui il paziente dipenda da trattamenti di sostegno vitale (LST), ha chiarito il requisito della 'dipendenza dai LST', collocandolo in un contesto più ampio, indipendentemente dalla sua complessità tecnica, invasività o dal diretto coinvolgimento di operatori sanitari.
In assenza di una legislazione specifica, le Sentenze della CC costituiscono la base per l'accesso attuale al *SMA*, ancora lungi dall'essere chiarito, con procedure ancora discordanti sul territorio nazionale.

Il ruolo del neurologo

Il ruolo del medico (il neurologo per quanto riguarda le malattie neurologiche) nei casi di *SMA* è sfaccettato, comprendendo aspetti clinici, etici e legali.
Per quanto riguarda il ruolo clinico, il medico (operante all'interno di una struttura pubblica del SSN) può essere coinvolto nella valutazione della condizione del paziente e dell'idoneità secondo la Sentenza 242/2019 della CC [4]. Questo include la conferma della diagnosi di "patologia irreversibile fonte di sofferenze fisiche o psicologiche" che il paziente "reputa intollerabili". Il medico deve anche comprendere la prognosi del paziente e verificare che la sua sofferenza non possa essere alleviata con altri mezzi. Inoltre, è essenziale valutare se il paziente sia "pienamente capace di prendere decisioni libere e informate" e sia "mantenuto in vita da *TSV*". Infine, è il medico che può valutare se il paziente sia in grado di auto-somministrarsi il farmaco letale, il che richiede la capacità di mantenere la motricità e la deglutizione. Considerato che disturbi mentali e/o sofferenza psicologica e/o deficit cognitivo possono compromettere la *capacità decisionale*, si consiglia al neurologo di collaborare con altri specialisti, come psichiatri, psicologi e neuropsicologi, per una valutazione più completa.
È necessario garantire che il paziente comprenda appieno le implicazioni della sua decisione. Ciò richiede una discussione approfondita sulla condizione del paziente, sulla prognosi e sulle opzioni alternative per assicurare che l'intento di porre fine alla propria vita sia "autonomamente e liberamente formatosi".
Il medico non è obbligato a prescrivere sostanze letali a meno che non sia direttamente coinvolto nella procedura di *SMA* su richiesta del paziente. In effetti, questa responsabilità ricade sul medico designato dal paziente, cui è stato affidato tale ruolo. Il medico che assiste nell'implementazione del *SMA* può essere supportato da altri professionisti per la preparazione dell'ambiente appropriato, del principio attivo letale e del dispositivo di somministrazione. Se il paziente lo consente, il medico dovrebbe cercare di collaborare strettamente con specialisti delle *CP*, considerando anche la gestione della fase del lutto.
Il ruolo etico del medico nel *SMA* è complesso, coinvolgendo un delicato equilibrio tra il rispetto dell'autodeterminazione del paziente e la gestione delle responsabilità professionali e delle convinzioni morali personali. Egli deve navigare in questo equilibrio rispettando i principi etici clinici di autonomia, beneficenza e non maleficenza [5]. È fondamentale preservare la riservatezza del paziente. Tuttavia, quando obblighi legali o etici sono in conflitto, i medici dovrebbero cercare consiglio da esperti di diritto e bioetica. I comitati etici locali per la pratica clinica, presenti in alcune regioni italiane, possono svolgere un ruolo importante nell'affrontare queste e altre questioni e conflitti [6,7].
Partecipare al *SMA* può comportare sfide professionali, inclusi potenziali conflitti con credenze morali personali o convinzioni etiche professionali. Alcuni medici possono provare disagio morale o subire critiche da colleghi o dal pubblico.

I medici devono rispettare requisiti legali specifici, come documentare la richiesta del paziente, garantire che tutti i criteri di idoneità siano soddisfatti e riferire il caso alle autorità competenti. Questo processo deve essere documentato meticolosamente per evitare problemi legali.
La pratica del *SMA* influenza la percezione, nella comunità medica generale, del ruolo dei medici.
Attualmente, i medici italiani sono guidati dal Codice di Deontologia Medica stabilito dalla Federazione Nazionale degli Ordini dei Medici Chirurghi e degli Odontoiatri (FNOMCEO), che afferma che "il medico, anche su richiesta del paziente, non deve effettuare né favorire atti finalizzati a provocarne la morte" (articolo 17) [8]. Tuttavia, a seguito della Sentenza CC 242/2019 [4], è in corso un dibattito sulle implicazioni per l'articolo 17 del Codice di Deontologa Medica. Per conciliare il Codice con la nuova realtà giuridica, è stata introdotta una modifica, secondo cui "la libera scelta del medico di agevolare, sulla base del principio di autodeterminazione dell'individuo, il proposito di suicidio autonomamente e liberamente formatosi [...] va sempre valutata caso per caso e comporta [...] la non punibilità del medico da un punto di vista disciplinare" [8]. I consigli disciplinari degli Ordini dei Medici saranno chiamati a valutare ogni caso specifico, per garantire che tutte le condizioni previste dalla Sentenza della CC siano rispettate. La FNOMCEO ha quindi deciso di lasciare i colleghi liberi di agire secondo la legge e la propria coscienza. Anche se i principi dell'articolo 17 rimangono immutati, la modifica indica uno spostamento nel divieto assoluto di atti che causino la morte su richiesta del paziente. Ciò è in linea con le disposizioni della CC che, al di fuori dell'ambito delimitato, ha ribadito che "l'incriminazione dell'aiuto al suicidio non è, di per sé, in contrasto con la Costituzione ma è giustificata da esigenze di tutela del diritto alla vita, specie delle persone più deboli e vulnerabili, che l'ordinamento intende proteggere evitando interferenze esterne in una scelta estrema e irreparabile, come quella del suicidio" [9].
L'adattamento di questo quadro etico ai cambiamenti giuridici in corso è un'area fondamentale di discussione all'interno della comunità medica italiana. Nella situazione attuale, la Sentenza n. 242/2019 della CC [4] afferma che non c'è "alcun obbligo" per i medici di assistere nel *SMA*, lasciando che sia una questione di coscienza individuale per i medici decidere se accogliere la richiesta di un paziente. Poiché il *SMA* non impone alcun obbligo di partecipazione agli operatori sanitari, non dovrebbe essere necessario stabilire l'obiezione di coscienza. Tuttavia, la CC ha riconosciuto che le istituzioni sanitarie pubbliche sono obbligate a rispondere alle richieste di *SMA*. Non ci sono problemi quando un neurologo accetta volontariamente di partecipare al *SMA* all'interno del rapporto medico-paziente. Il problema si presenta quando un neurologo è tenuto a partecipare alle procedure di *SMA* dovute dalla propria istituzione pubblica, come servire in un comitato per determinare l'idoneità al *SMA*. In tali casi, il neurologo può invocare la cosiddetta 'clausola di coscienza', che gli consente di obiettare sulla base dei principi costituzionali e dei codici etici in situazioni non regolate dalla legge. Tuttavia, se nessun medico della sanità pubblica fosse disposto a partecipare, si verificherebbe una situazione insostenibile, ostacolando il diritto del paziente al *SMA*. Il Comitato Nazionale per la Bioetica (CNB) italiano [10] ha sottolineato che l'obiezione di coscienza deve essere esercitata in modo sostenibile, garantendo che i servizi siano disponibili per tutelare i diritti dei pazienti nonostante le obiezioni. Questo principio potrebbe essere applicato in modo simile alla 'clausola di coscienza'.

Il contesto internazionale

Come evidenziato in una recente revisione [11], in Svizzera, nei Paesi Bassi e in alcuni stati degli Stati Uniti, si verifica un processo di *morte volontaria assistita* (*MVA*) nei pazienti neurologici con una frequenza solo inferiore a quella dei pazienti affetti da cancro. Negli studi esaminati, la condizione neurologica più frequente era la demenza, e l'*eutanasia* era dieci volte più comune del *SMA*.
La Tabella illustra la regolamentazione del *SMA* nei principali Paesi europei e non europei. Sono considerati i principali criteri di idoneità, inclusi quelli previsti dalla normativa attuale sul *SMA* in Italia.

Tabella. La regolamentazione del suicidio medicalmente assistito (*SMA*) nei principali Paesi europei e non europei. Vengono considerati i principali criteri normativi, inclusi quelli previsti dalla normativa italiana attuale. ACP = Advance Care Planning; NS = Non Specificato; TSV = Trattamento di Sostegno Vitale.

**Paese**	**Legge**	**Anno**	**Criteri normativi**							
			**Età (anni)**	**Volontarietà**	**Capacità decisionale**	**Condizione**	**Prognosi sfavorevole**	**TSV**	**Periodo di riflessione obbligatorio**	**Seconda valutazione / Commissione**
EUROPA										
Svizzera[a,b]	No	1942	≥18	Sì	Sì	NS, incluso disturbo psichiatrico	NS	NS	NS	NS/NS
Paesi Bassi[c]	*Termination of Life upon Request Act*	2002	≥12 (consenso genitoriale 12-16)	Sì	Sì, al momento della richiesta, incluso ACP	NS, incluso disturbo psichiatrico	NS	NS	NS	Sì (disturbo psichiatrico) / Sì
Belgio[a,c,d]	No	2002	Nessun limite	Sì	Sì, al momento della richiesta, incluso ACP	NS, incluso disturbo psichiatrico	NS	NS	NS	Sì (disturbo psichiatrico) / Sì
Lussemburgo[c]	*Right to Die with Dignity*	2009	≥16 (consenso genitoriale	Sì	Sì, al momento della	NS, incluso disturbo psichiatrico	NS	NS	NS	Sì (disturbo psichiatrico) / Sì

			16-18)		richiesta, incluso ACP					
Italia[a]	No	2019 CC	≥18	Sì	Sì	Patologia irreversibile fonte di sofferenze fisiche o psicologiche	NS	Sì	NS	Sì / Sì
Germania[a]	No	2020 CC	≥18	Sì	Sì	NS, escluso disturbo psichiatrico acuto	NS	NS	NS	NS
Spagna[c]	Sì	2021	≥18	Sì	Sì	Malattia grave o incurabile oppure una condizione cronica o invalidante che causa sofferenza intollerabile	NS	NS	15 giorni scritto	Sì / Sì
AMERICHE										
Oregon<br>Washington<br>D.C.<br>Maine	*Death with Dignity Act*	1994<br>2009<br>2016<br>2019	≥18	Sì	Sì	Terminale	<6 mesi	NS	2 giorni scritto<br>15 giorni orale	Sì / NS
Montana[a]	No	2009	NS	Sì	Sì	NS	NS	NS	NS	NS / NS

Vermont	*Patient Choice and Control at the EOL Act*	2013	≥18	Sì	Sì	Terminale	<6 mesi	NS	2 giorni scritto<br>15 giorni orale	Sì / NS
California<br>Colorado<br>Nuovo Messico	*End of Life Option Act*	2015<br>2016<br>2021	≥18	Sì	Sì	Terminale	<6 mesi	NS	15 giorni orale	Sì / NS
Hawaii	*Our Care, Our Choice Act*	2019	≥18	Sì	Sì	Terminale	<6 mesi	NS	6 giorni orale e scritto	Sì / NS
New Jersey	*Aid in Dying for the Terminalely Ill Act*	2019	≥18	Sì	Sì	Terninale	<6 mesi	NS	15 giorni orale e scritto	Sì / NS
Canada[c]	*Medical assistance in dying*	2016 (modificata 2021)	≥18	Sì	Sì, al momento della richiesta, incluso ACP	Grave, con prognosi sfavorevole (disturbo psichiatrico in valutazione)	Ragionevolmente prevedibile	NS	10 giorni scritto<br>90 giorni se assente 'morte ragionevolmente prevedibile' (es. disturbo psichiatrico)	Sì / NS
Colombia[a,c]	No	1997[2]<br>2022 Supreme Court	≥7 (consenso genitoriale 7-12)	Sì	Sì	Terminale o grave dipendenza	NS	NS	NS	NS / Sì
OCEANIA										
Victoria<br>Western Au	*Voluntary Assisted Dying*	2017<br>2019	≥18	Sì	Sì	Terminale	<6 mesi<br>(<12 mesi malattia	NS	9 giorni scritto	Sì / Sì

Tasmania[c]	*Act*	2021					neurodegenerativa)			
Nuova Zelanda[c]	*End of Life Choice Act*	2020	≥18	Sì	Sì	Terminale	<6 mesi	NS	No	Sì / Sì

Note:

a. In questi Paesi non esiste una legge per il *SMA*, ma è depenalizzato.

b. L'unico Paese in cui il *SMA* è consentito anche per i cittadini stranieri.

c. In questi Paesi l'eutanasia è legale.

d. Dal 2015 esteso ai minori; è richiesto il consenso dei genitori e una valutazione psicologica per i minori "non emancipati".

Un esempio della continua evoluzione del quadro normativo, che riflette i cambiamenti nella sensibilità etica riguardo alle questioni di *fine vita* in diversi Paesi, è rappresentato dagli sviluppi in Francia e nel Regno Unito, dove i processi legislativi sulle questioni di *fine vita* sono in corso. In Francia, nell'aprile 2023, la posizione maggioritaria di un'assemblea cittadina ("Convention Citoyenne sur la fin de vie") ha sostenuto la necessità di implementare sia il *SMA* sia l'eutanasia, sostenendo che né il solo *SMA* né la sola eutanasia rispondono a tutte le situazioni incontrate [12]. Nel novembre 2024, la House of Commons del Regno Unito ha votato a favore del "Terminally Ill (End of Life) Bill", che mira a legalizzare il *SMA*. Tuttavia, il disegno di legge deve ancora essere approvato dalla House of Lords e affrontare diversi ostacoli parlamentari prima di poter diventare legge [13].

Suicidio medicalmente assistito e malattie neurologiche

Si pongono diverse questioni peculiari per i pazienti che richiedono il *SMA* a causa di una patologia neurologica con *prognosi infausta*, così come per le persone per le quali una patologia irreversibile non neurologica (ad esempio, il cancro) costituisce la base della loro richiesta, ma che vivono contemporaneamente con una grave condizione neurologica. Gli individui diagnosticati con malattie neurodegenerative possono essere interessati e talvolta richiedere il *SMA*, ma si trovano a fronteggiare difficoltà riguardo alla tempistica. Infatti, l'aspettativa di vita media dopo l'insorgenza dei sintomi può essere compresa tra i 15 e i 30 anni, e quando la condizione soddisfa criteri per la richiesta di *SMA*, spesso si sviluppano sintomi psichiatrici e cognitivi severi che possono compromettere la *capacità decisionale* dell'individuo.

In condizioni che compromettono la *capacità decisionale* e, di conseguenza, la possibilità di fornire consenso, non è possibile richiedere il *SMA* (ai sensi della Sentenza della CC 242/19) e altre decisioni di fine vita (*decisioni di non trattamento*; *sedazione palliativa continua profonda*) ai sensi della Legge italiana 219/2017 [1].

La effettuazione del *SMA* nell'ambito della pianificazione anticipata delle cure (*Advance Care Plannng - ACP*) non è consentita dalla normativa italiana attuale. Tuttavia, potrebbe essere possibile fare riferimento a strutture sanitarie in paesi esteri. Per questo motivo, l'*ACP* dovrebbe essere particolarmente raccomandata in una fase relativamente precoce nei pazienti con malattie caratterizzate da un declino cognitivo progressivo. In questo caso, sarebbe possibile ricorrere successivamente a decisioni di *fine vita* in una fase in cui il consenso attuale non può essere espresso. Questa futura decisione di *fine vita* potrebbe includere il *SMA*, in accordo con i documenti di consenso e le linee guida dell'European Academy of Neurology sull'*ACP*, che suggeriscono ai neurologi di incoraggiare i pazienti a discutere i loro desideri riguardo alla *MVA* [14-16].

L'American Academy of Neurology (AAN) [17], dopo due anni di discussione, ha ritirato la propria posizione del 1998, che includeva la "forte opposizione dei suoi membri alla partecipazione a qualsiasi forma di suicidio assistito". Inoltre, l'AAN ha identificato la possibilità di posizioni a favore o contro la pratica del *SMA*, a discrezione del neurologo. L'AAN [18] ha enfatizzato l'importanza di incoraggiare il neurologo ad attuare percorsi per ottenere *direttive anticipate*.

Il *SMA* richiede l'auto-somministrazione che può essere problematica in molte malattie neurologiche a causa di compromissioni motorie e della deglutizione, creando una tensione con le leggi sulla disabilità che impongono assistenza e accesso equo alle cure sanitarie e all'autodeterminazione [19].

La posizione della SIN

La posizione della SIN è in accordo con il Mental Health Action Plan 2013-2020 adottato dall'Organizzazione Mondiale della Sanità [20]. Questo piano d'azione delinea la prevenzione del suicidio come una priorità, con l'obiettivo globale di ridurre il tasso di suicidi nei paesi del 10% entro il 2020. Nei Sustainable Development Goals per il 2030, il suicidio è un indicatore proposto per l'obiettivo di salute volto a ridurre la mortalità prematura per malattie non trasmissibili attraverso prevenzione e trattamento, promuovendo salute mentale e benessere [21]. La SIN sottolinea la necessità di migliorare le cure per i pazienti affetti da malattie potenzialmente letali in diverse fasi della malattia, affinché i neurologi padroneggino gli aspetti interdisciplinari/interprofessionali della medicina palliativa [Box]. I pazienti con dolore severo e/o altri sintomi significativi traggono beneficio dalla *CP* [14]. Inoltre, è essenziale che la legalizzazione del *SMA* debba avvenire solo dopo aver garantito l'accesso universale ai servizi di *CP* e ai farmaci appropriati, inclusi gli oppioidi per il dolore e la dispnea [22]. Quando la *CP* è offerta con un trattamento adeguato, il desiderio di morte può attenuarsi [10]. Ottimizzare le *CP*, ovviamente, non eliminerà tutte le richieste di *SMA*, ma potrebbe identificare e mirare a ciò che può essere modificato, inclusi i sintomi, il luogo di cura, il luogo di morte, massimizzando l'autonomia e la qualità della vita dei pazienti, qualunque sia il loro livello di funzionalità [17].

La SIN non ha espresso un'indicazione specifica riguardo al *SMA* nel Codice di Condotta Professionale [23]. Tuttavia, è dichiarato che devono essere rispettati i desideri del paziente riguardo agli approcci volti a prolungare la sopravvivenza e possibilmente garantire una "morte confortevole e dignitosa".

Infatti, la SIN ha approvato il proprio Codice Etico [24], che identifica anche i rapporti con le associazioni di pazienti e le famiglie, che sono monitorati da un organo di vigilanza, e fa riferimento al suddetto Codice di Condotta Professionale per i neurologi.

Nel contesto degli obiettivi da implementare nella conduzione delle cure, raccomandiamo ai neurologi di proporre la pianificazione condivisa delle cure ai loro pazienti in grado di prendere decisioni, in grado di esprimere i propri desideri. Considerato l'aumento del numero di pazienti neurologici che richiedono il *SMA*, la SIN ha pianificato di affrontare il tema per aumentare la consapevolezza all'interno della comunità neurologica e armonizzare le procedure tecniche e operative.

In presenza di sintomi refrattari, il neurologo può contribuire, ma anche eseguire autonomamente, la *sedazione palliativa continua profonda*, con il consenso del paziente. La somministrazione della *sedazione palliativa* può essere responsabilità dei neurologi se si considerano competenti a farlo, a condizione di ricevere la formazione necessaria, avere un ambiente ad hoc e la possibilità di richiedere assistenza ad altri specialisti, in primis ai palliativisti. Alcune malattie neurologiche progressive con *prognosi infausta*, così come condizioni di grave danno irreversibile al sistema nervoso che richiedono assistenza continua per garantire le funzioni vitali, rappresentano situazioni in cui i pazienti possono

richiedere consapevolmente il *SMA* a causa di sofferenze fisiche e/o psicologiche intollerabili e persistenti (*dolore totale*).

Nei casi di *SMA* richiesto da pazienti neurologici, una valutazione neurologica standardizzata è fondamentale per definire le condizioni di danno irreversibile al sistema nervoso ("patologia inguaribile" nella Sentenza della CC 242/19) [4]. Tuttavia, la valutazione del neurologo dovrebbe essere effettuata all'interno di un team interdisciplinare e interprofessionale, composto da esperti in *CP*, psicologia, neuropsicologia, psichiatria, anestesiologia-rianimazione, neuro-riabilitazione e dal Medico di Medicina Generale del paziente o l'equivalente in altri paesi.

Nella maggior parte delle malattie neurologiche progressive e irreversibili candidate al *SMA*, l'identificazione dei *TSV* appare un processo molto complesso. Il neurologo dovrebbe essere chiamato a esprimere il proprio giudizio sulla presenza e significato dei mezzi che possono essere interpretati come *TSV*. Di fronte a disabilità neurologiche gravi o gravissime, i neurologi dovrebbero valutare se l'assistenza fornita dal caregiver per il soddisfacimento dei bisogni vitali del paziente debba essere considerata *TSV*.

Considerando le diverse condizioni di danno neurologico, possono verificarsi situazioni di compromissione delle capacità cognitive, tali da apparire influenti negativamente sulla *capacità decisionale* del paziente. In quest'ultime condizioni, il riferimento a *direttive anticipate* può offrire al medico la consapevolezza di una sufficiente *capacità decisionale* del paziente al momento dell'espressione dei propri desideri. La ricerca di standard di riferimento per la valutazione della capacità nel contesto del *SMA* dovrebbe essere un obiettivo da perseguire all'interno della comunità scientifica neurologica.

L'uso di *direttive anticipate* per il *SMA*, lontano dall'essere fattibile nel contesto attuale, dovrebbe essere un punto di dibattito etico clinico all'interno della SIN per l'evoluzione futura della deontologia e della legge italiana, considerando quanto già accaduto in altri paesi. Allo stesso modo, il dibattito sull'*eutanasia* dovrebbe essere oggetto di discussione, in particolare per le specifiche condizioni in cui gravi condizioni neurologiche del paziente rendono impossibile o pericolosa l'attuazione pratica di un comportamento suicida [19].

La formazione per i neurologi su *CP* e discussioni di *fine vita* dovrebbe essere una priorità [Box], considerando la necessità attuale nel curriculum di laurea e post-laurea [25]. In Italia, similmente a quanto fatto da altre comunità scientifiche, in attesa dell'emanazione di una specifica misura legislativa richiesta dalla CC, i neurologi dovrebbero prendere decisioni sul *SMA* tenendo conto della sensibilità individuale di ciascuno e alle disposizioni emanate dagli organi competenti attivati in ciascun caso specifico. In conformità con l'opinione espressa dal CNB [10] e da altre posizioni internazionali, si ritiene che tale intervento legislativo debba includere una disposizione per l'obiezione di coscienza, consentendo ai neurologi di essere esentati dall'effettuare procedure e attività legate al *SMA*, ma non dall'erogare cure prima di esso. Le istituzioni sanitarie pubbliche o convenzionate dovrebbero essere tenute a garantire che le procedure delineate in tale ipotetica legge vengano attuate. A tal fine, si auspica che tali procedure siano riservate esclusivamente al sistema sanitario pubblico e incluse nei Livelli Essenziali di Assistenza (LEA) italiani [26]. Questo garantirebbe l'assenza di discriminazione nell'accesso al *SMA* e uguaglianza su tutto il territorio nazionale.

Riguardo al contesto in cui dovrebbe svolgersi l'atto finale di *SMA*, la proposta è principalmente per il domicilio del paziente, o in alternativa, in ambienti designati all'interno di strutture pubbliche o convenzionate, distinti da quelli in cui si svolgono altre attività diagnostiche e terapeutiche. Queste opzioni dovrebbero essere discusse con il paziente. I principi attivi, così come i dispositivi necessari per l'auto-somministrazione (che devono essere adattati alle capacità di ciascun paziente), dovrebbero essere completamente resi gratuiti dal SSN.

Data la nostra posizione sulla necessità di una legislazione, vorremmo infine concentrarci sull'argomento del 'pendio scivoloso', citato, tra le altre fonti, dal CNB [10], che percepiamo come un possibile ostacolo paradossale alla stessa definizione di una legge sulla *MVA*. La metafora del 'pendio scivoloso' mette in evidenza il rischio che una legislazione, che consente il *SMA* in casi specifici e ben definiti, possa espandersi oltre il suo intento originale. Pur comprendendo queste preoccupazioni, temiamo anche che tale argomento possa essere un ostacolo specioso all'autodeterminazione del paziente. Siamo fermamente convinti che l'argomento del 'pendio scivoloso' possa essere utile solo se promuove cautela nell'instaurare condizioni rigorose per il *SMA* e nell'introdurre metodi che possano garantire la correttezza di eventuali decisioni anticipate [Box]. A tal fine, sarebbe opportuno istituire comitati specifici per condurre revisioni regolari, monitorare le pratiche per garantire il rispetto dei criteri per *SMA*, segnalare eventuali problemi o abusi e discutere potenziali modifiche future alla luce di nuove situazioni.

Conclusioni

Mentre il dibattito sociale e politico attorno alla *MVA* rimane aspro e incessante, in Italia manca ancora una legislazione per la *MVA*, a differenza della sua espansione in Europa, America e Australia. Molte comunità si stanno attualmente confrontando con questioni relative alle cure di *fine vita* e nuove legislazioni sulla *MVA* sono state proposte da diversi partiti politici e associazioni. Con le tendenze demografiche, culturali e sociali che portano a una maggiore consapevolezza riguardo all'autonomia e all'autodeterminazione alla fine della vita, la *MVA* continuerà a crescere come una questione critica di salute pubblica. A questo proposito, la necessità di effettuare ricerca sull'impatto sui pazienti, sui medici, sui sistemi sanitari e sulle comunità sta diventando sempre più rilevante e urgente, così come il monitoraggio attento dell'aderenza a garanzie sostanziali e procedurali. I dati sulle pratiche di *MVA* sono limitati. Pertanto, è necessario raccogliere dati affidabili per valutare lo standard delle procedure e del percorso diagnostico riguardo al *SMA*. La SIN, tenendo conto dell'aumento del numero di individui affetti da patologie neurologiche che chiedono *MVA* e della necessità di una posizione condivisa, ha promosso una riflessione approfondita all'interno della comunità dei suoi membri. La SIN riconosce che la questione del *SMA* è estremamente controversa, con opinioni forti su entrambi i lati del dibattito, e sostiene la necessità di rispettare l'autonomia individuale e l'empatia verso coloro che sperimentano sofferenza. Allo stesso tempo, la SIN afferma il valore della vita umana e dell'importanza di creare comunità solidali che garantiscano dignità e proteggano le persone vulnerabili. Devono essere considerate anche le diverse culture e i valori religiosi. Per le ragioni articolate, proponiamo che il *SMA* dovrebbe essere legalizzato in casi selezionati, mentre investire in formazione, nel monitoraggio attento e nell'aggiornamento delle normative potrebbe prevenire qualsiasi espansione inappropriata delle

pratiche di *MVA* che i cittadini italiani potrebbero considerare immorali e incompatibili con la legge [Box]. La SIN riconosce che le basi morali possono evolversi in una società pluralista e in uno stato costituzionale, dove non sono le libertà personali ma le loro limitazioni a dover essere giustificate, proteggendo sia gli interessi della comunità sia quelli dell'individuo, soprattutto per coloro che si trovano in condizioni vulnerabili che ostacolano la formazione di una volontà consapevole e autentica. Qualsiasi futura espansione dell'accesso alla *MVA* dovrebbe essere vista come una risposta ragionevole a nuove esigenze cliniche, sociali e culturali, piuttosto che come una concessione indebita all'argomento del 'pendio scivoloso'.

Box. La posizione della SIN. *SMA*, suicidio assistito dal medico; *CP*, cure palliative.

La SIN riconosce che le malattie neurologiche:

- Sono la **seconda condizione più importante** per la richiesta di *SMA* dopo il cancro a livello mondiale
- Possono causare **sofferenza intollerabile** (fisica, psicologica e/o esistenziale)
- Possono presentare difficoltà in termini di **prognosi temporale**
- Possono coinvolgere la **capacità decisionale** e/o la **capacità comunicativa del paziente**
- Possono compromettere la **capacità funzionale del paziente di auto-somministrarsi sostanze**

La SIN afferma che:

- I neurologi dovrebbero riconoscere e discutere apertamente il **desiderio di morte assistita volontaria** con il paziente, identificare i fattori di rischio (es. depressione, isolamento, abilità limitate) e le possibili opzioni terapeutiche
- I **servizi di *CP*** dovrebbero essere disponibili per i pazienti con disturbi neurologici e le loro famiglie in tutte le regioni italiane e in diversi contesti (es. ricovero, ambulatorio, domicilio)
- I neurologi dovrebbero riconoscere e discutere apertamente la **richiesta di *SMA*** da parte del paziente quando il miglior trattamento sanitario disponibile (incluse le *CP*) non è efficace o è rifiutato dal paziente, in particolare nei casi con sofferenza spirituale/esistenziale
- La **decisione di partecipare o meno al *SMA*** dovrebbe essere lasciata al giudizio scrupoloso di ciascun neurologo, agendo per conto del proprio paziente
- È urgente una **legge italiana sul *SMA***, per garantire una procedura tutelata, trasparente e giusta
- È necessario un **monitoraggio continuo del *SMA*** per prevenire eventuali deviazioni nelle pratiche tra le diverse aree italiane o nel tempo

La SIN si impegna per:

- **Programmi di formazione** per i membri della SIN in materia di decisioni condivise, discussioni tempestive sul fine vita e pianificazione condivisa delle cure
- **Programmi di formazione incrociata** in Neurologia e *CP*, in particolare per i membri più giovani
- **Ricerca sulle cure neuropalliative**, con un focus sulla valutazione e il trattamento di pazienti con esigenze di cura complesse e sofferenza refrattaria.

Bibliografia

1.Solari A, Ticozzi N, Comi G et al (2025) Neurology and physician-assisted suicide: glossary of definitions and terminology. Neurol Sci. https://doi.org/10.1007/s10072-025-08064-3.

2.Radbruch L, Leget C, Bahr P, et al (2016) Euthanasia and physician-assisted suicide: A white paper from the European Association for Palliative Care. Palliat Med 30(2):104-16.

3.Codice penale e Leggi complementari. (2024) Ramacci F (ed). Giuffrè Francis Lefebvre. ISBN:9788828854517.

4.Corte Costituzionale (2019) Sentenza 242/2019. https://www.cortecostituzionale.it/documenti/download/doc/recent_judgments/Sentenza_n_242_del_2019_Modugno_en.pdf Ultimo accesso 7 Agosto 2024.

5.Beauchamp TL, Childress JF (2013) Principles of Biomedical Ethics. 8th Edition, Oxford University Press, New York.

6.Comitato Nazionale per la Bioetica (2017) I comitati per l'etica nella clinica. https://bioetica.governo.it/media/1395/p127_2017_i-comitati-etici-per-la-clinica_it.pdf. Ultimo accesso 14 Ottobre 2024.

7.Comitato Nazionale per la Bioetica (2023) Risposta Quesito del Ministero della Salute 2 Gennaio 2023. 24 febbraio 2023. https://bioetica.governo.it/media/4893/p150_2023_risposta-quesito-ministero-salute.pdf. Ultimo accesso 14 Gennaio 2025.

8.Federazione Nazionale degli Ordini dei Medici Chirurghi e degli Odontoiatri (FNOMCEO) (2020) Codice di Deontologia Medica. https://portale.fnomceo.it/codice-deontologico/. Ultimo accesso 14 Ottobre 2024.

9.Ufficio Stampa della Corte Costituzionale. Press release of 22 November 2019 (2019) End-of-life treatments: when assistance to suicide cannot be punished. https://www.cortecostituzionale.it/documenti/download/pdf/242_2019.pdf. Ultimo accesso 5 Ottobre 2024.

10.Comitato Nazionale per la Bioetica (2019) Riflessioni bioetiche sul suicidio medicalmente assistito. 18 luglio 2019. https://bioetica.governo.it/media/4310/vr__p135_2019_parere-suicidio-medicalmente-assistito.pdf. Ultimo accesso 20 Ottobre 2024.

11.Trejo-Gabriel-Galán JM (2021) Euthanasia and assisted suicide in neurological diseases: a systematic review. Neurologia (Engl Ed) S0213-4853(21)00090-6. doi: 10.1016/j.nrl.2021.04.016.

12.Delfraissy JF, Ruault C, Claeys A, Aubry R, Callies I (2024) End-of-life, participatory democracy, and legislative work in France. Lancet 13;403(10422):141. doi: 10.1016/S0140-6736(23)02580-1.

13.McKiernan J (2024). What happens next to the bill on assisted dying? Published 29 November 2024. BBC News. Ultimo accesso 20 Gennaio 2025.

14.Oliver DJ, Borasio GD, Caraceni A, et al (2016) A consensus review on the development of palliative care for patients with chronic and progressive neurological disease. Eur J Neurol 23:30–8.

15.Solari A, Giordano A, Sastre-Garriga J, et al (2020) EAN guideline on palliative care of people with severe, progressive Multiple sclerosis. Eur J Neurol 27(8):1510-29.

16.Van Damme P, Al-Chalabi A, Andersen PM, et al (2024) European Academy of Neurology (EAN) guideline on the management of amyotrophic lateral sclerosis in collaboration with European Reference Network for Neuromuscular Diseases (ERN EURO- NMD). Eur J Neurol 31: e16264.

17.Russell JA, Epstein LG, Bonnie RJ et al (2018) On behalf of the Ethics, Law, and Humanities Committee (a joint committee of the AAN, ANA, and CNS). Lawful physician-hastened death. AAN position statement. Neurology 90: 420-22.

18.Taylor LP, Besbris JM, Graf WD et al (2022) Clinical Guidance in Neuropalliative Care: An AAN Position Paper. Neurology 98:409-16.

19.Shavelson L, Pope TM, Battin MP, Ouellette A, Kluger B (2023) Neurologic Diseases and Medical Aid in Dying: Aid-in-Dying Laws Create an Underclass of Patients Based on Disability. Am J Bioeth 23(9): 5–15.

20.World Health Organization (2013). Mental health action plan 2013-2020.ISBN 9789241506021.

21.World Health Organization (2024) https://www.who.int/data/gho/data/themes/topics/sdg-target-3_4-noncommunicable-diseases-and-mental-health. Ultimo accesso 3 Ottobre 2024.

22.Provinciali L, Carlini G, Tarquini D, et al (2016) Need for palliative care for neurological diseases. Neurol Sci 37(10): 1581-7.

23.Società Italiana di Neurologia. Codice di Condotta Professionale https://www.neuro.it/web/eventi/NEURO/societa.cfm?p=codice_condotta_professionale. Ultimo accesso 5 Ottobre 2024.

24.Società Italiana di Neurologia. Codice Etico https://www.neuro.it/web/eventi/NEURO/societa.cfm?p=codice_etico. Ultimo accesso 5 Ottobre 2024.

25.Bombaci A, Di Lorenzo F, Pucci E, Solari A, Veronese S, Società Italiana di Neurologia–Società Italiana di Cure Palliative Intersociety Table (2024) Education needs in palliative care and advance care planning of Italian residents in neurology: an online survey. Eur J Neurol 31(9):e16376.

26.Istituto Superiore di Sanità https://www.iss.it/web/iss-en/essential-assistance-levels-LEA-Ultimo accesso 20 Ottobre 2024.
